# Intracranial Hemorrhage After Pfizer-BioNTech (BNT162b2) mRNA COVID-19 Vaccination: A Case Report

**DOI:** 10.7759/cureus.37747

**Published:** 2023-04-18

**Authors:** Kivanc Yangi, Doga D Demir, Ajlan Uzunkol

**Affiliations:** 1 Neurological Surgery, Prof. Dr. Cemil Tascioglu City Hospital, Istanbul, TUR; 2 Emergency Medicine, Prof. Dr. Cemil Tascioglu City Hospital, Istanbul, TUR

**Keywords:** bnt162b2 mrna, intracranial hemorrhage (ich), intracranial hemorrhage and sars-cov-2, tozinameran, covid-19 outbreak, bnt162b2 (pfizer-biontech)

## Abstract

The Coronavirus 2019 (COVID-19) pandemic has affected over 700 million people worldwide and caused nearly 7 million deaths. Vaccines currently developed or in development are the most effective tools for curbing the pandemic and mitigating its impacts. In Turkey, inoculation with the Pfizer-BioNTech COVID-19 vaccine (BNT162b2, also known as tozinameran) has been approved.

We report a 56-year-old female patient with underlying essential hypertension who experienced intracranial hemorrhage after receiving her first dose of tozinameran. The patient underwent immediate surgical evacuation of the hematoma, during which a left middle cerebral artery bifurcation aneurysm was macroscopically identified and clipped. The patient was pronounced deceased on the second postoperative day. This is the second case of intracranial hemorrhage following tozinameran administration caused by a ruptured middle cerebral artery bifurcation aneurysm. Upon analyzing the case, there might be a connection between the vaccine's potential immune-triggering effect on hemodynamic patterns and the rupture of the previously unknown cerebral aneurysm. However, these severe complications do not justify avoiding vaccines; further studies are needed. This study emphasizes the need for increased vigilance in patients with underlying systemic comorbidities who have recently been vaccinated and to share our insights into the potential relationship between tozinameran and intracranial hemorrhage.

## Introduction

Severe acute respiratory syndrome coronavirus-2 (SARS-CoV-2) has had global effects, causing both acute and chronic complications [1]. Medical authorities must continuously update their knowledge and establish guidelines for disease management. The vaccination program represents the most effective strategy for reducing the impact of the disease and ending the pandemic [2]. The European Medicines Agency has approved five vaccines, including the Pfizer-BioNTech COVID-19 vaccine (BNT162b2) [3]. Although there is a consensus that the benefits of vaccines outweigh their complications, some serious complications have arisen [4]. The most common side effects of vaccines encountered in daily practice include pain at the injection site, muscle pain, joint pain, headache, fever, and fatigue [2].

Existing literature suggests that the pathophysiology of COVID-19 may trigger cerebral aneurysm rupture; however, the precise relationship between COVID-19 and intracranial hemorrhage due to cerebral aneurysm rupture remains unclear [5]. To our knowledge, there is suspicion that anti-SARS-CoV-2 vaccines may cause intracranial hemorrhage, but this has not been officially recognized as a side effect of tozinameran by any authorities. Nevertheless, fatal and non-fatal cases have been reported in the literature [6]. We present a case of a 56-year-old female patient who experienced an intracerebral hemorrhage after receiving her first dose of tozinameran. With this report, we hope that our diagnostic and treatment process will serve as an example for managing patients with similar complaints following vaccination.

## Case presentation

**Figure 1:**
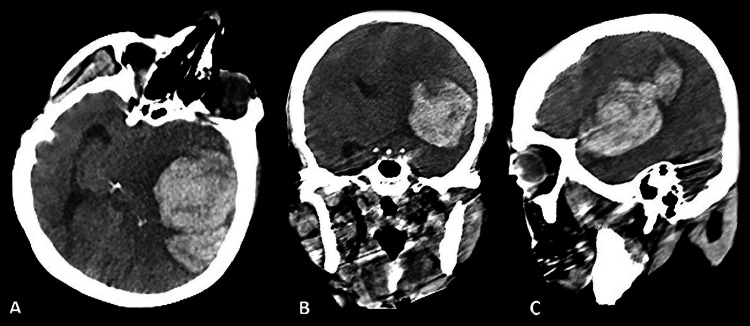
Preoperative Non-Contrast Enhanced Computed Tomography A: Preoperative cranial computed tomography axial scan showing a 6x6 cm hyperdense area in the left temporal lobe extending into the intraventricular space, with perilesional edema detected. B: Preoperative cranial computed tomography coronal scan revealing obliterated major sulci and a faint left lateral ventricle. A mass effect with a 9 mm midline shift was observed. C: Preoperative cranial computed tomography sagittal scan displaying a 6x6 cm hyperdense area, consistent with hemorrhage and perilesional edema detected.

A 56-year-old Caucasian female patient with underlying essential hypertension and hypothyroidism was admitted to the infectious diseases department for her first dose of the Pfizer-BioNTech mRNA COVID-19 vaccine in March 2023. The patient had no significant family history of chronic illnesses, does not use illicit drugs, and had no history of previous surgeries. The patient was a high-school math teacher. As we learned from her son, she was not using drugs unless she felt extremely discomfortable. Upon admission, her pre-vaccination physical examination revealed 110/70 mm Hg blood pressure, 36°C body temperature, and 129 mg/dL blood glucose level. Her initial physical examination of all systems was intact, and her Glasgow Coma Scale (GCS) score was 15 out of 15 (best possible score, her eyes were open spontaneously 4/4, and she obeyed commands 6/6 and spoke fluently and meaningfully 5/5; she was oriented to time, person, and place). Immediately after the vaccine injection, she became dizzy, experienced temporary cognitive dissociation, and exhibited decreased verbal responsiveness. The supervising physicians from the infectious diseases department referred the patient to the emergency department. Fifteen minutes after vaccination, the patient lost consciousness and demonstrated neither verbal nor motor responses to painful stimuli, with a GCS score of three out of 15 (worst possible score, no motor responses to painful stimuli 1/6, no verbal response 1/5, and no eye opening 1/4). There was no motor response to the painful stimuli in both the upper or lower extremities. Her pupils were dilated and unresponsive to light. The patient was intubated, and a non-enhanced cranial CT scan revealed a 6x6 cm hyperdense area in the left temporal lobe extending into the intraventricular space, accompanied by perilesional edema (Figure 1). Major sulci were obliterated, the left lateral ventricle appeared faint, and a mass effect with a 9 mm midline shift was observed. The neurosurgery department was consulted, and urgent surgical exploration was planned. Due to the emergent nature of the surgery, a contrast-enhanced cranial MRI or CT angiography could not be obtained. A hemorrhagic area extending to the cerebral cortex was observed during the surgery. Following transsylvian dissection, active arterial bleeding from an aneurysmatic dilatation was macroscopically identified at the bifurcation of the left middle cerebral artery. The aneurysm was clipped, and the hematoma was evacuated; thus, the middle cerebral artery bifurcation aneurysm was considered the primary cause. After the operation, the patient's vital signs were unstable, and she was transferred to the intensive care unit. Postoperative non-contrast-enhanced cranial computed tomography or CT angiography to verify the aneurysm could not be obtained due to the patient's unstable vital signs. The postoperative neurological examination revealed no response to painful stimuli and no pupillary reaction to light, with a GCS score of three out of 15 (worst possible score, no motor responses to painful stimuli 1/6, no verbal response 1/5, and no eye opening 1/4). The patient could not be extubated, and unfortunately, she was pronounced deceased on the second postoperative day.

## Discussion

To our knowledge, ten cases of intracranial hemorrhage after the Pfizer-BioNTech COVID-19 mRNA (BNT162b2) vaccine have been reported in the literature across four different studies [3,6-8]. In one case, ruptured arteriovenous malformation caused intracranial hemorrhage [3]. A ruptured cerebral aneurysm led to intracranial hemorrhage in five cases. Only one case was caused by a middle cerebral artery bifurcation aneurysm. In the other four cases, aneurysms were located at the internal carotid artery-posterior communicating artery, anterior communicating artery, and vertebral artery [7,8]. To our knowledge, this is the second case of intracranial hemorrhage following tozinameran injection caused by a ruptured middle cerebral artery bifurcation aneurysm.

In all reported cases, a couple of days elapsed before the intracranial hemorrhage occurred; however, in our case, it suddenly happened after the tozinameran injection. Female predominance has been reported in all cases similar to ours. Additionally, two cases were vaccinated for their second doses of tozinameran, while the other two were vaccinated for their first doses [7,8]. Furthermore, ten fatal cases following tozinameran injection were reported in the literature; five out of ten were due to intracranial hemorrhage. The incidence of intracranial hemorrhage among Japanese women who received tozinameran was disproportionately high, suggesting a possible causative relationship between intracranial hemorrhage and tozinameran [6].

The reason behind the aneurysm rupture should be discussed to better understand this issue. Inflammatory cascades in the cerebral aneurysm wall, primarily due to T-cells and macrophages, may be responsible for the rupture of cerebral aneurysms [9]. Abnormal immune responses are believed to play a critical role in the deadly cascade of COVID-19 [10]. Vaccination may activate and trigger abnormal innate and acquired immune responses, and these systemic immune responses may cause the rupture of an underlying arteriovenous malformation or cerebral aneurysm [11]. Additionally, systemic hyperinflammation, cytokine storm, and hyperviscosity induced by COVID-19 may be causes of vascular injury and trigger the rupture of malformed vessels. Conversely, inflammatory responses triggered by COVID-19 may alter hemodynamic patterns and cause a rupture of underlying arteriovenous malformations or cerebral aneurysms [12].

Intracerebral hemorrhage may also be caused by vasculitis, as reported in the literature in one case [13]. A recent study demonstrated that the frequencies of vasculitis within approximately two weeks after the first dose of Pfizer-BioNTech (BNT162b2) and Moderna (mRNA-1273) vaccines were 2.9% and 0.7%, respectively [14]. Another potential explanation for the etiology of intracranial hemorrhage following tozinameran injection is that immune thrombocytopenia after the administration of anti-SARS-CoV-2 vaccines may cause cerebral venous sinus thrombosis (CVST). CVST often results in fatal intracranial hemorrhage [15].

In our case, the patient had underlying untreated hypertension, mild hypothyroidism, and a family history of hypertension. She never experienced neurological symptoms requiring admission to the neurology or neurosurgery departments. The patient was admitted to the internal medicine department five months ago, and anti-hypertensive drugs were prescribed. However, she never used them, as we learned from her son and medical records. However, the patient's hemorrhage was located in an atypical position for hypertension-related hemorrhage. Consequently, the differential diagnosis includes amyloid angiopathy, CVST, previously unknown arteriovenous malformations, aneurysms, or neoplasms.

In this case, we believe that the reason for the intracranial hemorrhage was a rupture of the left middle cerebral artery bifurcation aneurysm. However, postoperative computed tomography and angiography could not be obtained due to the patient's unstable vitals.

In light of scientific data, vaccination remains the most potent and safe protection against COVID-19 disease, and it is not possible to definitively state that there is a direct causative relationship between intracranial hemorrhage and COVID-19 vaccination. While these complications do not hinder administering second or subsequent immunization doses, physicians should be aware of these potentially fatal situations that may arise after vaccination.

This privileged case report emphasizes a potentially brutal relationship between the tozinameran and intracranial hemorrhage. We want to warn our colleagues about the potential side effects of COVID-19 (Appendix 1). According to our recommendations, care should be taken while examining the patients before getting vaccinated for COVID-19. If there is an underlying chronic disease, such as an unknown cerebral aneurysm, it may cause crucial events. If a patient shows an unwanted side effect after the vaccine injection, such as loss of consciousness, dizziness, or drowsiness, they should be closely monitored risk of intracerebral hemorrhage should always be kept in mind. Needed imagings have to be done immediately.

There are some limitations in our study. Preoperative high-quality CT scans and CT angiography could not be obtained. An intraoperative picture of the aneurysm and the postoperative head CT or CT angiography could not be obtained because of the patient's unstable vitals.

## Conclusions

This case cannot demonstrate a direct relationship between the COVID-19 Pfizer-BioNTech vaccination and intracerebral hemorrhage. Based on our findings, we highly recommend examining the patients thoroughly before vaccination and paying close attention to those with underlying untreated essential hypertension, especially in the first few hours after vaccination. While examining the patients before the vaccination, the physician should always remember that there might be an unknown underlying disease. Patients who exhibit neurological or cognitive symptoms after vaccination should be closely monitored, and necessary imaging or interventions should be promptly performed to prevent devastating outcomes. Upon analyzing the case, there might be a connection between the vaccine's potential immune-triggering effect on hemodynamic patterns and the rupture of the previously unknown cerebral aneurysm. However, these severe complications do not justify avoiding vaccines; further studies are needed. In order to hypothesize the relationship between intracranial hemorrhage and the anti-COVID-19 vaccines, more studies involving more patients are needed.

## Appendices

Appendix 1

Patient's perspectives through her son: '' Yesterday, when I talked to my mother on the phone, she said she would get her first dose of the COVID-19 vaccine. How would I know that this was the last speech between us? She was never so attached to the hospitals; she was rarely admitted to the hospitals. During the hot pandemic days, we could not convince her to get her vaccinations, but suddenly on these days, she decided to take care of her health more. My whole family is shocked. I think this bleeding was because of uncontrolled hypertension; she never got the anti-hypertensive drugs the cardiologist prescribed. However, it may also be caused by the vaccination. Please publish this and inform the world about the potential side effect of this vaccine. I don't want to see any other families mourning.''
